# 5-Methyl-7,8,9,10-tetra­hydro­cyclo­hepta­[*b*]indol-6(5*H*)-one

**DOI:** 10.1107/S1600536811016229

**Published:** 2011-05-07

**Authors:** R. Archana, E. Yamuna, K. J. Rajendra Prasad, A. Thiruvalluvar, R. J. Butcher

**Affiliations:** a PG Research Department of Physics, Rajah Serfoji Government College (Autonomous), Thanjavur 613 005, Tamilnadu, India; b Department of Chemistry, Bharathiar University, Coimbatore 641 046, Tamilnadu, India; c Department of Chemistry, Howard University, 525 College Street NW, Washington, DC 20059, USA

## Abstract

In the title mol­ecule, C_14_H_15_NO, the dihedral angle between the benzene and pyrrole rings is 1.99 (12)°. The cyclo­heptene ring adopts a slightly distorted boat conformation.

## Related literature

For the inter­est and importance of indole derivatives, see: Csomós *et al.* (2007 ▶). For pyrido-fused cyclo­hept[*b*]indole alkaloids, see: Bennasar *et al.* (1997 ▶). For crystallographic studies of cyclo­hept[*b*]indoles, see: Archana *et al.* (2010 ▶).
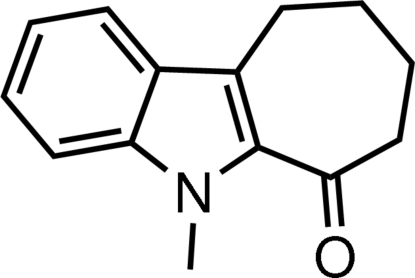


## Experimental

### 

#### Crystal data

C_14_H_15_NO
*M* *_r_* = 213.27
Orthorhombic, 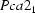

*a* = 8.6999 (2) Å
*b* = 14.1805 (3) Å
*c* = 9.1392 (3) Å
*V* = 1127.49 (5) Å^3^
*Z* = 4
Cu *K*α radiation
μ = 0.62 mm^−1^
*T* = 295 K
0.47 × 0.35 × 0.20 mm

#### Data collection

Oxford Diffraction Xcalibur Ruby Gemini diffractometer
Absorption correction: multi-scan (*CrysAlis PRO*; Oxford Diffraction, 2010 ▶) *T*_min_ = 0.803, *T*_max_ = 1.000
1184 measured reflections
1184 independent reflections
1148 reflections with *I* > 2σ(*I*)
*R*_int_ = 0.020

#### Refinement

*R*[*F* ^2^ > 2σ(*F* ^2^)] = 0.037
*wR*(*F* ^2^) = 0.106
*S* = 1.07
1184 reflections
147 parameters
1 restraint
H-atom parameters constrained
Δρ_max_ = 0.14 e Å^−3^
Δρ_min_ = −0.13 e Å^−3^

### 

Data collection: *CrysAlis PRO* (Oxford Diffraction, 2010 ▶); cell refinement: *CrysAlis PRO*; data reduction: *CrysAlis PRO*; program(s) used to solve structure: *SHELXS97* (Sheldrick, 2008 ▶); program(s) used to refine structure: *SHELXL97* (Sheldrick, 2008 ▶); molecular graphics: *ORTEP-3* (Farrugia, 1997 ▶); software used to prepare material for publication: *PLATON* (Spek, 2009 ▶).

## Supplementary Material

Crystal structure: contains datablocks global, I. DOI: 10.1107/S1600536811016229/hg5032sup1.cif

Structure factors: contains datablocks I. DOI: 10.1107/S1600536811016229/hg5032Isup2.hkl

Supplementary material file. DOI: 10.1107/S1600536811016229/hg5032Isup3.cml

Additional supplementary materials:  crystallographic information; 3D view; checkCIF report

## Figures and Tables

**Fig. 1.**
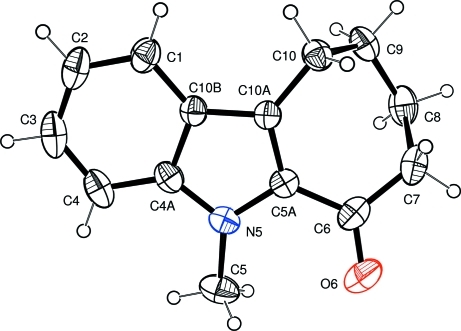
The molecular structure of the title compound, showing the atom-numbering scheme and displacement ellipsoids drawn at the 30% probability level. H atoms are shown as small spheres of arbitrary radius.

## supplementary crystallographic information

### Comment

Indole derivatives condensed with different heterocycles are physiologically active compounds found in abundance in materials such as pharmaceuticals, alkaloids and potential therapeutic agents (Csomós *et al.*, 2007). Ervitsine and Ervatamine (Bennasar *et al.*, 1997) were important class of pyrido fused cyclohept[*b*]indole alkaloids. Recently we have reported crystallographic studies for some cyclohept[*b*]indoles in our laboratory (Archana *et al.*, 2010).

The molecular structure of the title compound, with atomic numbering scheme, is shown in Fig. 1. In the title molecule, C_14_H_15_NO, the dihedral angle between the benzene and pyrrole rings is 1.99 (12)°. The cycloheptene ring adopts a slightly distorted boat conformation.

### Experimental

To a solution of 7,8,9,10-tetrahydrocyclohepta[*b*]indol-6(5*H*)-one (0.199 g, 0.001 mol) in 5 ml acetone added powdered KOH (0.280 g, 0.005 mol) in ice cold condition. After few minutes methyl iodide (0.13 ml, 0.002 mol) was added drop by drop with vigorous stirring and the reaction mixture was stirrired for 15 min at room temperature. Benzene was added to the reaction mixture and insoluble materials are removed by filtration. The benzene solution was washed with saturated NaCl solution, dried by using Na_2_SO_4_ and evaporation yielded the title compound (0.191 g, 90%). This was recrystallized from benzene and ethyl acetate mixture.

### Refinement

Owing to the absence of any anamalous scatterers in the molecule, the Friedel pairs were merged. The absolute structure in the present model have been chosen arbitrarily. H atoms were positioned geometrically and allowed to ride on their parent atoms, with C—H = 0.93 - 0.97 Å and *U*_iso_(H) = 1.2 - 1.5 times *U*_eq_(C).

### Crystal data

**Table:**

C_14_H_15_NO	*D*_x_ = 1.256 Mg m^−^^3^
*M**_r_* = 213.27	Melting point: 338 K
Orthorhombic, *P**c**a*2_1_	Cu *K*α radiation, λ = 1.54184 Å
Hall symbol: P 2c -2ac	Cell parameters from 2006 reflections
*a* = 8.6999 (2) Å	θ = 4.8–73.4°
*b* = 14.1805 (3) Å	µ = 0.62 mm^−^^1^
*c* = 9.1392 (3) Å	*T* = 295 K
*V* = 1127.49 (5) Å^3^	Chunk, pale-yellow
*Z* = 4	0.47 × 0.35 × 0.20 mm
*F*(000) = 456	

### Data collection

**Table:**

Oxford Diffraction Xcalibur Ruby Gemini diffractometer	1184 independent reflections
Radiation source: Enhance (Cu) X-ray Source	1148 reflections with *I* > 2σ(*I*)
graphite	*R*_int_ = 0.020
Detector resolution: 10.5081 pixels mm^-1^	θ_max_ = 73.6°, θ_min_ = 6.0°
ω scans	*h* = 0→10
Absorption correction: multi-scan (*CrysAlis PRO*; Oxford Diffraction, 2010)	*k* = 0→17
*T*_min_ = 0.803, *T*_max_ = 1.000	*l* = 0→11
1184 measured reflections	

### Refinement

**Table:**

Refinement on *F*^2^	Hydrogen site location: inferred from neighbouring sites
Least-squares matrix: full	H-atom parameters constrained
*R*[*F*^2^ > 2σ(*F*^2^)] = 0.037	*w* = 1/[σ^2^(*F*_o_^2^) + (0.0678*P*)^2^ + 0.0651*P*] where *P* = (*F*_o_^2^ + 2*F*_c_^2^)/3
*wR*(*F*^2^) = 0.106	(Δ/σ)_max_ = 0.001
*S* = 1.07	Δρ_max_ = 0.14 e Å^−^^3^
1184 reflections	Δρ_min_ = −0.13 e Å^−^^3^
147 parameters	Extinction correction: *SHELXL97* (Sheldrick, 2008), Fc^*^=kFc[1+0.001xFc^2^λ^3^/sin(2θ)]^-1/4^
1 restraint	Extinction coefficient: 0.018 (2)
Primary atom site location: structure-invariant direct methods	Absolute structure: see *Refinement* section in *Supplementary materials*
Secondary atom site location: difference Fourier map	

### Special details

**Table:**

Geometry. Bond distances, angles *etc*. have been calculated using the rounded fractional coordinates. All su's are estimated from the variances of the (full) variance-covariance matrix. The cell e.s.d.'s are taken into account in the estimation of distances, angles and torsion angles
Refinement. Refinement of *F*^2^ against ALL reflections. The weighted *R*-factor *wR* and goodness of fit *S* are based on *F*^2^, conventional *R*-factors *R* are based on *F*, with *F* set to zero for negative *F*^2^. The threshold expression of *F*^2^ > 2σ(*F*^2^) is used only for calculating *R*-factors(gt) *etc*. and is not relevant to the choice of reflections for refinement. *R*-factors based on *F*^2^ are statistically about twice as large as those based on *F*, and *R*- factors based on ALL data will be even larger.

### Fractional atomic coordinates and isotropic or equivalent isotropic displacement parameters (Å^2^)

**Table:**

	*x*	*y*	*z*	*U*_iso_*/*U*_eq_	
O6	0.5677 (3)	0.05292 (17)	0.4636 (4)	0.1141 (10)	
N5	0.4250 (2)	0.18603 (13)	0.6610 (2)	0.0514 (5)	
C1	0.4932 (3)	0.42768 (16)	0.7515 (3)	0.0607 (8)	
C2	0.3719 (3)	0.4495 (2)	0.8403 (4)	0.0792 (10)	
C3	0.2600 (4)	0.3822 (2)	0.8755 (4)	0.0826 (10)	
C4	0.2661 (3)	0.2923 (2)	0.8220 (3)	0.0686 (9)	
C4A	0.3895 (2)	0.26879 (16)	0.7301 (2)	0.0512 (7)	
C5	0.3392 (3)	0.09825 (18)	0.6805 (4)	0.0791 (10)	
C5A	0.5637 (3)	0.19791 (14)	0.5882 (2)	0.0471 (6)	
C6	0.6360 (4)	0.12408 (16)	0.4995 (3)	0.0637 (9)	
C7	0.7989 (3)	0.13870 (18)	0.4529 (3)	0.0669 (9)	
C8	0.9072 (3)	0.1731 (2)	0.5724 (3)	0.0713 (9)	
C9	0.9058 (3)	0.2786 (2)	0.6046 (3)	0.0647 (8)	
C10	0.7621 (3)	0.33087 (14)	0.5528 (3)	0.0533 (6)	
C10A	0.6154 (2)	0.28910 (14)	0.6081 (2)	0.0431 (5)	
C10B	0.5048 (2)	0.33542 (14)	0.6960 (2)	0.0458 (6)	
H1	0.56661	0.47293	0.72821	0.0728*	
H2	0.36341	0.51021	0.87808	0.0950*	
H3	0.17945	0.39907	0.93704	0.0989*	
H4	0.19098	0.24819	0.84567	0.0823*	
H5A	0.25845	0.10790	0.75052	0.1184*	
H5B	0.29541	0.07936	0.58858	0.1184*	
H5C	0.40722	0.04986	0.71507	0.1184*	
H7A	0.83814	0.07958	0.41476	0.0803*	
H7B	0.80020	0.18409	0.37351	0.0803*	
H8A	0.88223	0.13988	0.66208	0.0856*	
H8B	1.01113	0.15535	0.54543	0.0856*	
H9A	0.91596	0.28753	0.70940	0.0776*	
H9B	0.99487	0.30708	0.55857	0.0776*	
H10A	0.76018	0.33072	0.44669	0.0640*	
H10B	0.76828	0.39599	0.58476	0.0640*	

### Atomic displacement parameters (Å^2^)

**Table:**

	*U*^11^	*U*^22^	*U*^33^	*U*^12^	*U*^13^	*U*^23^
O6	0.131 (2)	0.0782 (13)	0.133 (2)	−0.0248 (14)	0.030 (2)	−0.0505 (16)
N5	0.0472 (9)	0.0535 (9)	0.0534 (10)	−0.0053 (7)	−0.0045 (8)	0.0057 (8)
C1	0.0620 (13)	0.0556 (12)	0.0644 (14)	0.0153 (10)	−0.0049 (12)	−0.0035 (11)
C2	0.0837 (19)	0.0764 (16)	0.0774 (18)	0.0354 (15)	0.0030 (16)	−0.0123 (16)
C3	0.0671 (15)	0.111 (2)	0.0696 (17)	0.0405 (18)	0.0133 (15)	0.0049 (17)
C4	0.0442 (12)	0.0964 (17)	0.0652 (15)	0.0115 (12)	0.0043 (11)	0.0162 (15)
C4A	0.0408 (10)	0.0643 (12)	0.0486 (12)	0.0048 (9)	−0.0069 (9)	0.0089 (10)
C5	0.0732 (17)	0.0691 (14)	0.095 (2)	−0.0234 (13)	−0.0062 (17)	0.0152 (16)
C5A	0.0508 (11)	0.0484 (10)	0.0421 (10)	0.0017 (8)	−0.0035 (9)	0.0048 (9)
C6	0.0855 (18)	0.0514 (12)	0.0541 (14)	0.0054 (11)	−0.0011 (13)	−0.0046 (10)
C7	0.0838 (18)	0.0654 (13)	0.0514 (13)	0.0252 (12)	0.0133 (13)	0.0021 (11)
C8	0.0639 (14)	0.0833 (17)	0.0668 (16)	0.0278 (13)	0.0048 (13)	0.0131 (15)
C9	0.0421 (11)	0.0857 (16)	0.0663 (15)	0.0022 (11)	0.0033 (11)	0.0069 (14)
C10	0.0538 (11)	0.0519 (9)	0.0542 (13)	−0.0005 (9)	0.0045 (10)	0.0087 (9)
C10A	0.0436 (10)	0.0438 (8)	0.0418 (10)	0.0050 (7)	−0.0036 (8)	0.0043 (8)
C10B	0.0421 (10)	0.0506 (10)	0.0448 (11)	0.0083 (7)	−0.0055 (8)	0.0029 (8)

### Geometric parameters (Å, °)

**Table:**

O6—C6	1.216 (4)	C10A—C10B	1.415 (3)
N5—C4A	1.368 (3)	C1—H1	0.9300
N5—C5	1.462 (3)	C2—H2	0.9300
N5—C5A	1.388 (3)	C3—H3	0.9300
C1—C2	1.367 (4)	C4—H4	0.9300
C1—C10B	1.407 (3)	C5—H5A	0.9600
C2—C3	1.401 (4)	C5—H5B	0.9600
C3—C4	1.366 (4)	C5—H5C	0.9600
C4—C4A	1.403 (3)	C7—H7A	0.9700
C4A—C10B	1.413 (3)	C7—H7B	0.9700
C5A—C6	1.466 (3)	C8—H8A	0.9700
C5A—C10A	1.381 (3)	C8—H8B	0.9700
C6—C7	1.494 (4)	C9—H9A	0.9700
C7—C8	1.523 (4)	C9—H9B	0.9700
C8—C9	1.525 (4)	C10—H10A	0.9700
C9—C10	1.529 (4)	C10—H10B	0.9700
C10—C10A	1.495 (3)		
			
C4A—N5—C5	123.97 (19)	C2—C3—H3	119.00
C4A—N5—C5A	108.26 (17)	C4—C3—H3	119.00
C5—N5—C5A	127.26 (19)	C3—C4—H4	121.00
C2—C1—C10B	118.7 (2)	C4A—C4—H4	121.00
C1—C2—C3	121.3 (3)	N5—C5—H5A	109.00
C2—C3—C4	121.8 (3)	N5—C5—H5B	109.00
C3—C4—C4A	117.8 (3)	N5—C5—H5C	110.00
N5—C4A—C4	130.8 (2)	H5A—C5—H5B	109.00
N5—C4A—C10B	108.16 (16)	H5A—C5—H5C	109.00
C4—C4A—C10B	121.1 (2)	H5B—C5—H5C	109.00
N5—C5A—C6	123.5 (2)	C6—C7—H7A	108.00
N5—C5A—C10A	109.49 (18)	C6—C7—H7B	108.00
C6—C5A—C10A	127.0 (2)	C8—C7—H7A	108.00
O6—C6—C5A	122.2 (3)	C8—C7—H7B	108.00
O6—C6—C7	120.1 (3)	H7A—C7—H7B	107.00
C5A—C6—C7	117.7 (2)	C7—C8—H8A	108.00
C6—C7—C8	115.3 (2)	C7—C8—H8B	108.00
C7—C8—C9	116.6 (2)	C9—C8—H8A	108.00
C8—C9—C10	115.0 (2)	C9—C8—H8B	108.00
C9—C10—C10A	113.67 (19)	H8A—C8—H8B	107.00
C5A—C10A—C10	127.19 (19)	C8—C9—H9A	109.00
C5A—C10A—C10B	106.73 (17)	C8—C9—H9B	109.00
C10—C10A—C10B	126.05 (18)	C10—C9—H9A	108.00
C1—C10B—C4A	119.44 (18)	C10—C9—H9B	108.00
C1—C10B—C10A	133.24 (19)	H9A—C9—H9B	108.00
C4A—C10B—C10A	107.31 (17)	C9—C10—H10A	109.00
C2—C1—H1	121.00	C9—C10—H10B	109.00
C10B—C1—H1	121.00	C10A—C10—H10A	109.00
C1—C2—H2	119.00	C10A—C10—H10B	109.00
C3—C2—H2	119.00	H10A—C10—H10B	108.00
			
C5—N5—C4A—C4	−4.8 (4)	N5—C5A—C6—O6	12.5 (4)
C5—N5—C4A—C10B	174.5 (2)	N5—C5A—C6—C7	−167.6 (2)
C5A—N5—C4A—C4	−177.2 (2)	C10A—C5A—C6—O6	−164.4 (3)
C5A—N5—C4A—C10B	2.2 (2)	C10A—C5A—C6—C7	15.4 (4)
C4A—N5—C5A—C6	−178.7 (2)	N5—C5A—C10A—C10	177.9 (2)
C4A—N5—C5A—C10A	−1.3 (2)	N5—C5A—C10A—C10B	−0.1 (2)
C5—N5—C5A—C6	9.3 (4)	C6—C5A—C10A—C10	−4.8 (4)
C5—N5—C5A—C10A	−173.3 (2)	C6—C5A—C10A—C10B	177.2 (2)
C10B—C1—C2—C3	−0.4 (5)	O6—C6—C7—C8	−134.4 (3)
C2—C1—C10B—C4A	1.6 (3)	C5A—C6—C7—C8	45.7 (3)
C2—C1—C10B—C10A	−177.2 (2)	C6—C7—C8—C9	−81.6 (3)
C1—C2—C3—C4	−0.6 (5)	C7—C8—C9—C10	19.9 (3)
C2—C3—C4—C4A	0.3 (5)	C8—C9—C10—C10A	54.1 (3)
C3—C4—C4A—N5	−179.7 (3)	C9—C10—C10A—C5A	−57.7 (3)
C3—C4—C4A—C10B	1.0 (4)	C9—C10—C10A—C10B	119.9 (2)
N5—C4A—C10B—C1	178.60 (19)	C5A—C10A—C10B—C1	−179.6 (2)
N5—C4A—C10B—C10A	−2.3 (2)	C5A—C10A—C10B—C4A	1.5 (2)
C4—C4A—C10B—C1	−2.0 (3)	C10—C10A—C10B—C1	2.4 (4)
C4—C4A—C10B—C10A	177.16 (19)	C10—C10A—C10B—C4A	−176.58 (19)
